# Elective Treatment Strategy for Knee Prosthetic Joint Infection

**DOI:** 10.7759/cureus.76171

**Published:** 2024-12-22

**Authors:** Tatsuaki Matsumoto, Kazuya Kaneda, Shu Kobayashi, Kengo Harato, Takao Kodama

**Affiliations:** 1 Orthopaedics, Keio University, School of Medicine, Tokyo, JPN; 2 Orthopaedics, Saitama Medical Center, Saitama, JPN

**Keywords:** arthroplasty, debridement antibiotics and implant retention, prosthetic joint infection, total knee arthroplasty, unicompartmental knee arthroplasty

## Abstract

Introduction: Prosthetic joint infection (PJI) is a complication, rarely encountered in daily clinical practice, but its treatment is frequently unsuccessful. In this report, we describe the treatment strategy used at our hospital, which has provided stable therapeutic results.

Methods: We conducted a retrospective analysis of infections following knee arthroplasty at our hospital between April 2005 and December 2022.

Results: Post-total knee arthroplasty (TKA) infection occurred in 26 of 2132 patients (1.2%), and post-unicompartmental knee arthroplasty (UKA) infection occurred in six of 842 patients (0.7%); the difference was not significant (p=0.22). Infection occurred ≤3 months postoperatively in 13 patients with TKA and three with UKA, and ≥4 months postoperatively in 13 patients with TKA and three with UKA. Recurrent infection was observed in five patients with TKA and no patients with UKA. We did not conduct surgery for patients with PJI until causative bacteria were identified. We performed debridement, antibiotics, and implant retention (DAIR), with debridement of the synovium and insert replacement. Irrigation or drain placement was not conducted. The appropriate antibiotic was administered at a sufficient dose for a sufficiently long period, and the transfer of antibiotics into the bone marrow was not considered. Although some cases of recurrence were noted, treatment was successful in all cases, with no instances of obvious implant loosening or medullary lesions.

Conclusions: In the absence of implant loosening, the post-arthroplasty infection can be controlled by elective debridement surgery and selective administration of the optimal antibiotic.

## Introduction

In the United States, over 1 million knee arthroplasties are conducted every year for conditions such as osteoarthritis of the knee and osteonecrosis [1,2]. A variety of complications have been reported to occur after arthroplasty, including prosthetic joint infection (PJI), deep venous thrombosis, and perioperative mortality [3,4]. Postoperative infection is a complication rarely encountered in everyday clinical practice, and treatment is frequently unsuccessful [5]. Debridement, antibiotics, and implant retention (DAIR) is considered the best treatment modality for patients presenting with early PJI [6]. The advantage of a DAIR procedure for a patient is that the well-fixed implant remains, with lower morbidity and medical costs [7]. Regarding DAIR, it is controversial as to when to perform it after infection. In this report, we describe our hospital’s treatment strategy for elective DAIR.

## Materials and methods

Patients and methods

We conducted a retrospective analysis of infections following knee arthroplasty (2132 cases of total knee arthroplasty (TKA) and 842 cases of unicompartmental knee arthroplasty (UKA)) conducted in our hospital between April 2005 and December 2022. The inclusion criteria were all patients who underwent TKA or UKA surgery at our hospital during the above period; cases with positive joint fluid cultures were classified as infections, and no exclusion criteria were set. 

In the surgical procedure for TKA, cefazolin (1 g) was administered intravenously before surgery. If there was no renal dysfunction, cefazolin was administered up to three times every eight hours after surgery. The surgeries were conducted by seven surgeons. The implant was attached with cement on both the femoral and tibial sides in all cases, and a tourniquet was used only when intraoperative cementing was in progress. After treatment, weight-bearing was permitted from the day after surgery, and a Jones bandage was applied for three days postoperatively [8], after which range of motion exercises were started.

In the surgical procedure for UKA, cefazolin (1 g) was administered intravenously before the start of surgery, which was performed by six surgeons. The implant was attached with cement on the tibial side; on the femoral side, either it was attached with cement or a cementless implant was used. A tourniquet was used in the same manner as in TKA, and the post-treatment was also the same.

In both TKA and UKA, normal saline pulse lavage and a pressurized carbon dioxide lavage system were used in all cases to keep the bone as dry as possible so that no bone marrow fluid was included in the cement.

The causative bacteria, medical history, and treatment methods of patients diagnosed with postoperative infection were analyzed.

Treatment strategy

On the same day that a patient with a suspected infection presented to our hospital, blood samples were collected, and imaging tests and arthrocentesis were conducted (Table 1). The white blood cell (WBC) count, C-reactive protein (CRP), and procalcitonin levels were measured in the collected blood, and other tests were performed during the primary surgery. Two blood culture sets were also collected. In the imaging tests, plain radiographs were compared with previous images to evaluate the presence of implant loosening (e.g., deterioration of radiolucent lines). If necessary, additional computed tomography or magnetic resonance imaging were performed, but these are often difficult to assess because of artifacts and halation. In arthrocentesis, the most important of these tests, the aspirated fluid is cultured in blood culture bottles. Gram-stained smear tests were performed, and the synovial fluid was investigated for synovial fluid crystals, glucose level, WBC count, and esterase activity and [9,10] α-defensin activity [9,11] as indices of WBC activity.

**Table 1:** Progress of the treatment strategy for infection after knee arthroplasty POD, postoperative day; TKA, total knee arthroplasty

Day 0	Days 3-5	~Day 7 (POD 3)	Approximately one month postoperatively	Six months postoperatively	
Presentation	Culture results; sensitivity determination	-	-	-	
Blood collection; imaging; arthrocentesis	Debridement; intravenous antibiotic treatment started	Start same rehabilitation program as after TKA	Switch from IV to oral antibiotics	Consider discontinuing oral antibiotics	

**Figure 1:**
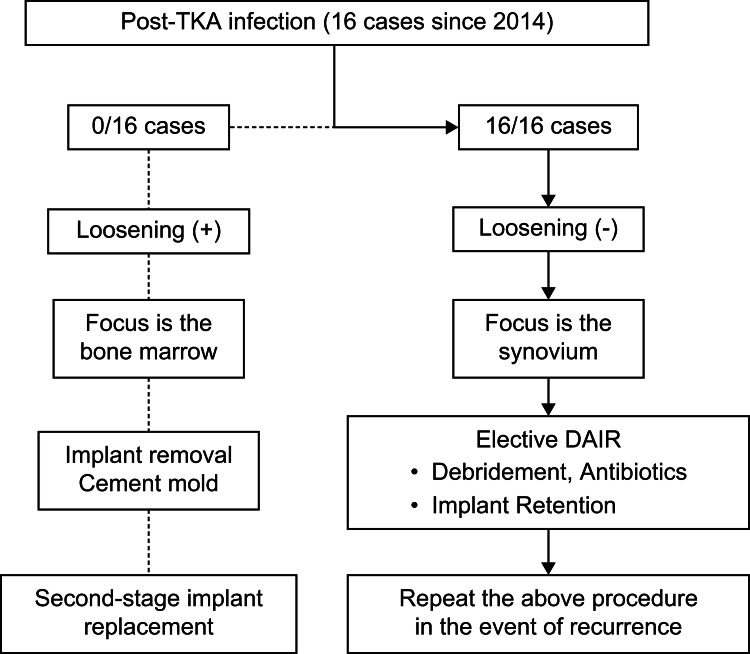
Flowchart of the treatment strategy for infection after TKA Since 2014, in the absence of implant loosening, we assumed that the infection was in the synovium, and we performed DAIR without removing the implant and planned to repeat DAIR if the infection recurs. TKA, total knee arthroplasty; DAIR, debridement, antibiotics, and implant retention

In the case of TKA, until 2013, the implant was removed, and surgery was performed using a semi-spacer in patients who exhibited repeated infections, regardless of implant loosening, if osteomyelitis had occurred alongside PJI. There were no cases of post-UKA infection before 2013. Since 2014, however, the synovium has been considered as the main source of postoperative infections that progress to osteomyelitis only in intractable cases. Hence, if there was no implant loosening, surgery was not performed in patients with PJI until the bacteria were identified by synovial fluid culture and the results of antibiotic sensitivity testing had returned (Figure 1). If the infection was indeed in the synovium, DAIR was performed electively without removing the metal implant in patients with TKA and UKA. The insert was changed during DAIR. Irrigation or drain placement (used in cases until 2013) was not conducted, and antibiotics were not added to the lavage solution.

Rehabilitation followed the same program as for primary knee arthroplasty. The antibiotic administration route was switched from intravenous to oral at about one month postoperatively once blood test data were improved (ideally to CRP ≤0.5), fever was resolved, and local knee symptoms were improved. Antibiotic administration was discontinued when the absence of recurrent infection was confirmed after approximately six months. If infection recurred during the postoperative course, the treatment described above was repeated.

Imaging was performed postoperatively and compared with previous postoperative images to reveal obvious implant loosening. Since 2014, implant loosening did not occur in any patient with PJI. Once the bacteria had been identified and the results of antibiotic sensitivity testing had returned, the implant was removed, and the cement mold was replaced as the first-stage procedure. Subsequently, the most appropriate antibiotic was administered to suppress the infection before replacing the implant as the second stage of treatment.

Statistical analysis

The χ2 test and Welch’s t-test were used for statistical analysis, with p<0.05 regarded as significant. The statistical software used was GraphPad Prism 8 (MDF, Boston, USA).

Ethics statement

This retrospective study was conducted with approval from the ethics committee of our institution (approval number: 22-35). As this study does not involve any additional invasiveness to patients, a public notification opt-out system has been implemented.

## Results

Post-TKA infection occurred in 26 of 2132 patients (1.2%) and post-UKA infection in six of 842 patients (0.7%), with a statistically no significant difference between them (p=0.22). The mean age at infection was 73.9±9.5 years (54-87 years) after TKA and 71.0±9.7 years (58-79) years after UKA. Notably, 10 of the 26 patients with TKA (38%) and four of the six patients with UKA (67%) had diabetes mellitus. However, previous steroid use and smoking history could not be traced. Infection occurred ≤3 months postoperatively in 13 patients with TKA and three patients with UKA, and ≥4 months postoperatively in 13 patients with TKA and three patients with UKA. The causative bacteria were Staphylococcus sp., including methicillin-resistant Staphylococcus aureus (MRSA), in most of the patients with TKA, and most post-UKA infections were also caused by Staphylococcus sp. (Table 2). The average waiting period until surgery was 4.4±0.8 days. There were no cases of sepsis until the waiting period for surgery.

**Table 2:** Bacteria causing TKA and UKA postoperative infections MRSA, methicillin-resistant Staphylococcus aureus; MRSE, methicillin-resistant Staphylococcus epidermidis; TKA, total knee arthroplasty; UKA, unicompartmental knee arthroplasty

Causative bacteria	TKA	UKA	Remarks
Staphylococcus sp., including two cases of MRSE	9	3	-
MRSA	2	-	-
Streptococcus sp.	7	1	-
Candida albicans	2	-	Both patients had tinea pedis
Peptostreptococcus	2	-	Oral/respiratory organ microbiota
Serratia marcescens	1	-	Oral/fecal microbiota
Enterococcus faecalis	1	-	Gut microbiota
Finegoldia magna	1	-	Skin microbiota
Escherichia coli	1	1	Gut microbiota
Granulicatella adicens	-	1	Oral/fecal microbiota

Since 2014, when we changed our treatment strategy, 16 cases of infection were noted, with recurrent infection occurring in five patients with TKA (31%) and in no patients with UKA (0%), showing no statistically significant difference between the two groups (p=0.15). The causative bacteria in the five patients with TKA were methicillin-sensitive Staphylococcus aureus in three cases, Staphylococcus lugdunensis in one, and Candida albicans in one. The patients with Candida albicans infection had tinea pedis. Revision surgery was performed twice in three cases, three times in one, and four times in one. In cases where reoperation was performed twice or three times, DAIR was conducted during the second and third surgeries as well. The patient who underwent four revision surgeries showed signs of skin necrosis and also underwent two local skin flap formation operations. Two cases of MRSE infection were successfully controlled using DAIR and postoperative administration of vancomycin hydrochloride.

## Discussion

There have been no previous reports of performing DAIR electively after TKA. Post-arthroplasty infection is reported to occur in 1-2% of patients [12], with a reported recovery rate of 60-80% [13,14]. Infection control methods include the use of antibiotic-containing cement in primary arthroplasty [15,16]. The addition of iodine to lavage solutions has been used in spinal surgery to reduce infection rates [17]. Intramedullary antibiotic administration before the start of surgery has also been reported to reduce infection rate [18]. Other studies have described a two-stage revision surgery using an antibiotic-containing cement mold [19] for cases of infection; in cases of PJI that do not involve the knee, continuous local antibiotic perfusion reportedly suppresses the infection [20,21].

Improvements in implants over time and the establishment of cementing techniques, such as the pressurized carbon dioxide lavage system [22,23], have resulted in no case of implant loosening at our hospital since 2014. Revision surgery reportedly has a high rate of reinfection [24,25], and it is important to use a reliable cementing technique to ensure that infection-induced implant loosening does not occur.

The second key factor in the treatment of PJI is the choice of antibiotic, and it is important to administer a sufficient dose of an antibiotic, to which the causative bacteria are sensitive, for a sufficiently long period. No previous studies have provided evidence for distinguishing between osteomyelitis and synovitis in the treatment of PJI, and no previous studies have reported on the use of DAIR after the identification of the causative bacteria and the determination of antibiotic sensitivity. Vancomycin (VCM), which is used to treat infections, including MRSA, reportedly does not transfer well to the bone marrow; however, when osteomyelitis lacks signs of implant loosening [26], we emphasize that our main focus is synovitis, and we do not find it necessary to consider antibiotic transfer to the bone marrow. Some studies recommend empirical antimicrobial therapy, except in patients with MRSA who require VCM treatment [27], but the indiscriminate use of empirical antibiotic treatment from the day of presentation should be avoided because of the possibility that it may cause the generation of resistance; this is another reason not to start antibiotic administration until sensitivity has been determined. Currently, there is no consensus on the duration of antibiotic treatment, but studies have found no difference between postoperative administration for three or six months, and we believe that it should be continued for a minimum of three months [28]. If sepsis is diagnosed based on blood culture tests performed on the day of presentation, immediate emergency surgery and same-day empirical antibiotic treatment must be considered.

Because some patients with suspected infection may actually have crystal deposition arthritis, DAIR should not be conducted until PJI has been diagnosed, the causative bacteria have been identified, and its sensitivity has been determined. Recurrent infection after initial DAIR reportedly occurs in 20-40% of cases, and the recurrence rate in our hospital after elective DAIR is approximately at the same level, which is a far from poor record [29]. We also recommend conducting DAIR after determining antibiotic sensitivity to avoid premature surgery. Staphylococcus infections accounted for three of the 11 cases of recurrent infection in this study, and as it has previously been reported that patients with Staphylococcus infections have poorer outcomes; caution is required [29,30].

We found that even recurrent infections can be suppressed by elective DAIR and appropriate antibiotic treatment, following the protocol described above. The absence of Tsukayama classification type 4 late-onset infections in the cases at our hospital means that we no longer need to carry out revision arthroplasty; however, it is important to educate patients to seek medical attention as soon as symptoms appear [5].

Through the careful use of cementing techniques in primary surgery and the treatment strategy described above, should infection occur, we have been able to avoid highly invasive revision surgery. Although some patients have developed recurrent infections, our treatment has been successful in all cases, with no obvious implant loosening or medullary lesions to date.

Limitation

This study did not include cases with implant loosening or cases that did not improve with DAIR, so additional analysis is required to determine how to respond to such cases. It is necessary to increase the number of samples and establish a treatment method for PJI in the future.

## Conclusions

PJI can be controlled by elective DAIR and appropriate antibiotic treatment. Treatment outcomes will not change even if DAIR is not performed urgently. Future prospective studies on the elective use of DAIR are warranted.
